# Supplementary material for: Overfitting Bayesian Mixture Models with an Unknown Number of Components
Source: PLoS One. 2015 Jul 15;10(7):e0131739. doi: 10.1371/journal.pone.0131739 (PMC4503697; doi:10.1371/journal.pone.0131739)
Supplement: S1 Table — Parameter summaries are included for n = 100 and n = 200 for all non-empty components for Sim 1 to 4. 95% Bayesian credible intervals are included for all estimates. K^¯0 defines the number of non-empty groups in the configuration considered in that row, and is annotated by an asterisk when this is correct. The parameter estimates corresponding to this configuration which contain the true value are similarly identified with an asterisk. (PDF) [file pone.0131739.s010.pdf]

Sim	n	$\underline{\hat{K}}_0$	k	$\hat{\pi}$ (95% CI)	$\hat{\mu}$ (95% CI)	$\hat{\sigma}^2$ (95% CI)
1	100	2	1	0.54 (0.44, 0.63)	5.56 (4.72, 6.47)	9.41(6.26,14.44)
1	100	2	2	0.46 (0.37, 0.56)	14.95 (14.52, 15.39)	2.25(1.51,3.37)
1	100	3*	1	0.47 (0.37, 0.57)*	14.95 (14.53, 15.37)*	2.27 (1.53, 3.37)
1	100	3*	2	0.34 (0.19, 0.46)*	7.11 (6.54, 7.71)*	2.09 (1.23, 3.66)
1	100	3*	3	0.19 (0.10, 0.34)*	2.71 (0.80, 4.93)*	9.47 (4.31, 18.04)
1	200	3*	1	0.48 (0.41, 0.55)*	14.87 (14.62, 15.12)*	1.49 (1.11, 1.99)
1	200	3*	2	0.33 (0.26, 0.41)*	7.22 (6.91, 7.54)*	1.47 (1.01, 2.12)
1	200	3*	3	0.19 (0.13, 0.26)*	1.89 (1.04, 3.08)*	5.02 (2.69, 9.28)
2	100	2	1	0.55 (0.44, 0.67)	-0.68 (-1.14, 0.05)	2.29 (1.20, 4.89)
2	100	2	2	0.45 (0.33, 0.56)	9.03 (8.13, 9.99)	5.48 (1.90, 9.28)
2	100	3*	1	0.51 (0.39, 0.62)*	-0.81 (-1.23, -0.42)*	1.69 (1.09, 2.69)
2	100	3*	2	0.38 (0.29, 0.48)*	9.79 (9.26, 10.3)*	2.31 (1.43, 3.82)
2	100	3*	3	0.11 (0.04, 0.21)*	3.85 (1.76, 5.48)*	3.95 (1.42, 10.54)*
2	200	3*	1	0.53 (0.46, 0.60)*	-0.93 (-1.13 ,-0.73)*	0.98 (0.73, 1.32)
2	200	3*	2	0.35 (0.28, 0.42)*	9.88 (9.54, 10.23)*	1.80 (1.23, 2.65)
2	200	3*	3	0.12 (0.07, 0.18)	4.25 (3.14, 5.32)*	3.77 (1.67, 8.39)*
3	100	1	1	1.00 (1.00, 1.00)	1.08 (0.61, 1.55)	5.79 (4.41, 7.62)
3	100	2*	1	0.41 (0.08, 0.76)*	1.49 (0.07, 5.32)*	11.5 (5.70, 21.90)
3	100	2*	2	0.59 (0.24, 0.92)*	0.94 (0.40, 1.41)*	1.66 (0.72, 3.75)*
3	200	2*	1	0.61 (0.43, 0.77)*	1.01 (0.75,1.28)*	1.24 (0.72,1.89)
3	200	2*	2	0.39 (0.23, 0.57)*	1.46 (0.59,2.39)*	12.85 (8.41, 19.91)
4	100	2	1	0.59 (0.43, 0.71)	5.89 (5.60, 6.19)	0.85 (0.53, 1.35)
4	100	2	2	0.41 (0.29, 0.57)	9.43 (8.51, 10.06)	2.09 (1.09, 4.11)
4	200	3*	1	0.65 (0.57, 0.72)*	5.99 (5.78, 6.20)*	1.01 (0.74, 1.37)*
4	200	3*	2	0.33 (0.25, 0.41)*	9.95 (9.54, 10.30)*	1.31 (0.79, 2.24)*
4	200	3*	3	0.02 (0.00, 0.07)*	13.81 (8.96, 19.04)	20.81 (7.28, 55.38)
